# Interleukin-1 and cancer progression: the emerging role of interleukin-1 receptor antagonist as a novel therapeutic agent in cancer treatment

**DOI:** 10.1186/1479-5876-4-48

**Published:** 2006-11-10

**Authors:** Anne M Lewis, Sheelu Varghese, Hui Xu, H Richard Alexander

**Affiliations:** 1 Surgical Metabolism Section, Surgery Branch, Center for Cancer Research, National Cancer Institute, Bethesda, Maryland, USA; 2 Howard Hughes Medical Institute-National Institutes of Health Research Scholars Program, 4000 Jones Bridge Road, Chevy Chase, Maryland, USA; 3 Department of Surgery and The Greenebaum Cancer Center, University of Maryland Medical Center, Baltimore, Maryland, USA

## Abstract

The tumor microenvironment consists of tumor, immune, stromal, and inflammatory cells which produce cytokines, growth factors, and adhesion molecules that promote tumor progression and metastasis. Of particular interest in this setting is interleukin-1 (IL-1), a pleiotropic cytokine with numerous roles in both physiological and pathological states. It is known to be up regulated in many tumor types and has been implicated as a factor in tumor progression via the expression of metastatic and angiogenic genes and growth factors. A number of studies have reported that high IL-1 concentrations within the tumor microenvironment are associated with a more virulent tumor phenotype. Solid tumors in which IL-1 has been shown to be up regulated include breast, colon, lung, head and neck cancers, and melanomas, and patients with IL-1 producing tumors have generally bad prognoses. The exact mechanisms by which IL-1 promotes tumor growth remain unclear, though the protein is believed to act via induction of pro-metastatic genes such as matrix metalloproteinases and through the stimulation of adjacent cells to produce angiogenic proteins and growth factors such as VEGF, IL-8, IL-6, TNFα, and TGFβ. The IL-1 receptor antagonist (IL-1ra) is a naturally occurring inhibitor to IL-1 and acts by binding to the IL-1 receptor without activating it. The protein has been shown to decrease tumor growth, angiogenesis, and metastases in murine xenograft models. Our focus in this review is to summarize the known data on the role of IL-1 in tumor progression and metastasis and the use of IL-1 inhibition as a novel therapeutic approach in the treatment of solid organ malignancies.

## Background

The tumor microenvironment consists of tumor, immune, stromal, and inflammatory cells all of which produce cytokines, growth factors, and adhesion molecules that may promote tumor progression and metastases. All intimately interact with one another and play an important role in inflammatory and pro-angiogenic processes and promote tumor cell proliferation. Interestingly, there is an association between chronic inflammation and tumor development or progression; 15% of all cancers are attributed to inflammatory etiologies [1]. The normal physiologic inflammatory response occurs upon tissue injury due to mechanical stimuli, infectious agents, or chemical irritation. Inflammatory cells communicate with fibroblasts and stromal cells via cytokines and chemokines and under the usual circumstances, reparative inflammation is limited. However, chronic pathological inflammation is mediated via the continuing presence of a persistent stimulus, such as tumor cells, and the resulting prolonged inflammatory cytokine exposure has the potential to promote tumor growth through the induction of angiogenesis, DNA damage, and other events favorable to tumor invasion and metastases, as reviewed in reference [2].

Cytokines regulate growth, trafficking, signalling, and differentiation of both stromal and tumor cells. The cytokines produced by cancer cells function to create optimal growth conditions within the tumor microenvironment, while the cytokines secreted by stromal cells may influence the behavior of malignant cells [3]. Cytokines induced by hypoxia, a hallmark feature present in progressive cancers, include vascular endothelial growth factor (VEGF), tumor necrosis factor (TNF), IL-1, and IL-6, as reviewed in reference [2]. TNF is a mediator of acute and chronic inflammation and has been detected in colon, breast, prostate and ovarian carcinomas as well as lymphomas and leukemias [4]. TNF, IL-6, and IL-8 can directly or indirectly promote tumor growth via induction of VEGF expression [5,6]. Of particular interest is IL-1, a pleiotropic cytokine with numerous roles in both physiological and pathological states. It is known to be up regulated in many tumor types and has been implicated as a factor in tumor progression via the expression of metastatic and angiogenic genes and growth factors. A number of studies have reported that high IL-1 concentrations within the tumor microenvironment are associated with a more virulent tumor phenotype. The exact mechanism through which IL-1 exerts its proliferative and angiogenic effects is unknown; it is postulated that interactions within the tumor microenvironment are believed an important component.

IL-1 receptor antagonist (IL-1ra) is an approved treatment for patients with rheumatoid arthritis and is being investigated as a potential novel therapeutic in cancer treatment. This naturally occurring protein has been shown to decrease tumor growth, angiogenesis, and metastases in murine xenograft models. There are other agents that are capable of inhibiting the inflammatory and tumor promoting effects of IL-1 such as anti-interleukin-1 monoclonal antibodies, the soluble IL-1 receptor type II, interleukin-1β-converting enzyme inhibitors, and IL-1 cytokine traps. Such potential therapeutic agents are currently being applied to the treatment of rheumatoid arthritis, but additional studies are necessary to determine their ability as novel therapy in cancer treatment. Our focus here is on the metastatic and angiogenic properties of IL-1 and the inhibition of this pro-inflammatory cytokine as a novel therapeutic approach in the treatment of solid organ malignancies.

## Biochemistry of IL-1

Three proteins comprise the IL-1 family, two of which are agonists, IL-1α and IL-1β; the third is IL-1 receptor antagonist (IL-1ra). IL-1α and IL-1β are derived from different genes but are functionally similar, and both bind to the same receptor [7-9]. Although they exhibit similar biological activities, IL-1α and IL-1β differ in the manner in which they are processed and secreted. IL-1α is localized in the cytosol or cell membrane and is believed to regulate the intracellular environment [8,9]. In contrast, IL-1β is first cleaved by interleukin-1β-converting enzyme (ICE) to its mature active form and then secreted extracellularly. Patients with infectious or inflammatory conditions exhibited elevated plasma concentrations of IL-1β but not IL-1α, suggesting the systemic role of IL-1β [9,10].

There are two IL-1 receptors, the biologically active IL-1 receptor type I (IL-1RI) and the IL-1 receptor type II (IL-1RII). Both are members of the immunoglobulin superfamily and contain structurally similar IL-1 binding sites [8]. IL-1RI is expressed on most cell types, preferentially binds IL-1α, and is responsible for the signal transduction of IL-1, whereas IL-1RII acts as an antagonist. Found within B lymphocytes, neutrophils, and monocytes, IL-1RII preferentially binds IL-1β and contains a shorter cytoplasmic segment. This decreases its signal transduction ability, attenuating the response to IL-1. Due to these antagonistic effects, IL-1RII is referred to as a decoy IL-1 receptor, resulting in its potential as a novel therapeutic agent [8,11]. Upon binding of IL-1 to either receptor type, the IL-1R accessory protein (IL-1RAcP) is recruited to the complex [11]. The IL-1RAcP facilitates signal transduction via the recruitment of kinases and activator and intracellular proteins. Without IL-1RAcP, IL-1 remains capable of binding IL-1RI but signal transduction is unable to occur [11,12]. This important mechanism affects the efficacy of therapeutics such as IL-1ra that attempt to prevent the IL-1 and IL-1RI interaction.

## IL-1 in cancer

IL-1 is a pluripotent cytokine responsible for normal physiological roles ranging from the induction of vascular permeability and fever during sepsis to the increased secretion of additional cytokines in autoimmune diseases. Other roles of IL-1 include production and release of prostaglandins, pituitary hormones, and collagenases. IL-1 also stimulates the immune system to boost production of lymphocytes. Therefore, an important balance exists between the beneficial and harmful effects of IL-1. It can negatively impact the vascular, endocrine, connective tissue, immune, haematopoietic, and central nervous systems, rendering it an important therapeutic target in a number of pathological conditions including rheumatoid arthritis, atherosclerosis, diabetes mellitus type I, inflammatory bowel disease and other autoimmune disorders, Alzheimer's disease, leukaemia, and solid tumors [8,13,9].

**Figure 1:**
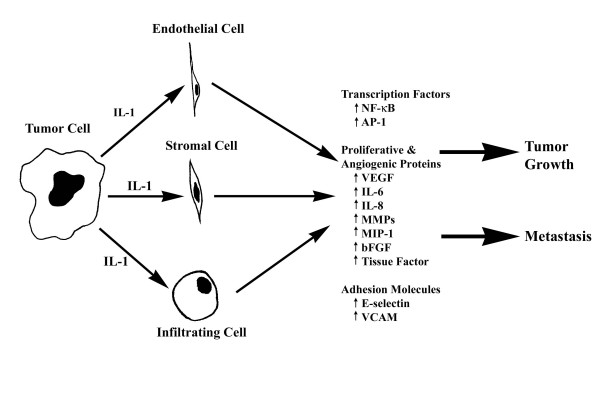
**Proposed model of how IL-1 indirectly alters tumor growth and metastatic potential in vivo**. In the tumor microenvironment IL-1 has local effects on host infiltrating cells that result in production of proangiogenic and prometastatic mediators.

Cancer cells directly produce IL-1 or can induce cells within the tumor microenvironment to do so [14]; studies have documented constitutive IL-1β protein production in human and animal cancer cell lines including sarcomas and ovarian and transitional cell carcinomas [9]. Solid tumors in which IL-1β has been shown to be up regulated include breast, colon, lung, head and neck cancers, and melanomas, and patients with IL-1β producing tumors have generally bad prognoses [6,15-17]. Elaraj *et al *[15] evaluated melanoma, non-small cell carcinoma, colon, and squamous cell cancer cell lines for the gene expression of IL-1α and IL-1β via real time quantitative reverse transcriptase PCR and found several of these lines to exhibit significantly increased copy numbers of IL-1α or IL-1β (Table 1). The expression patterns of IL-1 vary; it is expressed in an autocrine or paracrine fashion [18]. IL-1 exhibits autocrine behavior by stimulating the tumor cell itself to invade and proliferate, or it can exert paracrine effects on stromal cells in the microenvironment. The exact mechanisms by which IL-1 promotes tumor growth remain unclear, though the protein is believed to act primarily indirectly (Figure 1). IL-1 induces expression of metastatic genes such as matrix metalloproteinases (MMP) and stimulates nearby cells to produce angiogenic proteins and growth factors such as VEGF, IL-8, IL-6, TNFα, and tumor growth factor beta (TGFβ) [9,19-21]. Recent studies have determined the necessity of IL-1 in tumor growth, metastasis, and angiogenesis [7,9].

**Table 1:** Results of IL-1α or IL-1β mRNA expression in several tumor cell lines evaluated by quantitative RT-PCR.

**Human tumor cell lines**	IL-1α*	IL-1β*
SMEL (melanoma)	**3,267**	0
WIDR (colon cancer)	1	**122,932**
H2030 (NSCLC)	0	**29,084**
PMEL (melanoma)	35	67
SL-2 (squamous cell cancer)	140	0

* Gene expression is expressed as copy number per 10^5 ^copies of β-actin; copy number > 1,000/10^5 ^copies β-actin is in boldface.

### Tumor growth and metastases

The ability of IL-1 to induce the expression of angiogenic factors such as VEGF and IL-8 is believed to promote tumor growth and metastasis. Tumor metastasis is a multi-step process requiring numerous factors such as interactions between the tumor cell and its microenvironment [7,19,22]. Sawai *et al *[22] evaluated the role of IL-1 in metastatic and non-metastatic pancreatic cancer cell lines. Metastatic lines demonstrated increased IL-1RI expression compared to non-metastatic lines and exposure to IL-1α resulted in increased α6- and β1- integrin subunit expression, whereas IL-1α exposure in non-metastatic lines had no effect [22]. Additionally, IL-1α induced adhesion and invasion into laminin in metastatic pancreatic cell lines but not in non-metastatic lines. These studies highlighted the importance of IL-1α in cell adhesion and invasion into extracellular matrix proteins, and studies by Voronov *et al *[7] complement findings regarding the importance of IL-1 in metastasis. They demonstrated that IL-1α/IL-1β knockout mice failed to develop solid tumors following injection of melanoma cells and exhibited significantly improved survival compared to the wild-type animals, which died due to lung metastases [7]. Additional findings established that IL-1β was predominately responsible for the increased metastatic potential of the tumors. These studies indicate that presence of the IL-1RI receptor in cancer cells and IL-1 in the tumor microenvironment are important factors in tumor cell angiogenesis and adhesion and invasion into extracellular matrix [7,22].

### Angiogenesis

**Figure 2:**
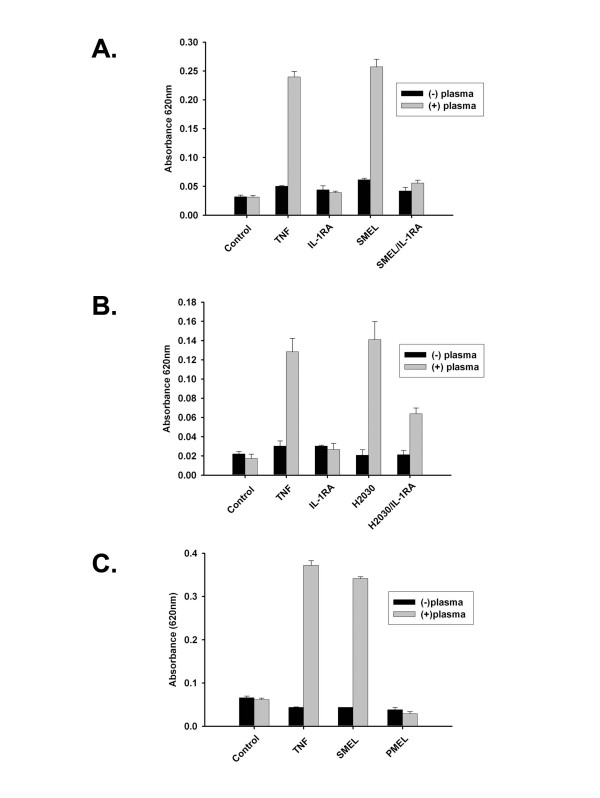
**Induction of endothelial cell monolayer permeability by tumor conditioned supernatant in an IL-1-dependent manner**. Permeability across functional endothelial cell monolayers was determined by measuring absorbance at 620 nm 1 hour following supernatant exposure. Induction of permeability by SMEL (A) and H2030, a lung non-small-cell carcinoma, (B) was completely or partially blocked by co incubation with 10 mg/mL IL-1Ra. PMEL did not induce permeability under identical experimental conditions (C). Tumor necrosis factor (TNF) at 10 ng/mL served as positive control. Columns, mean; bars, SE.

The ability of IL-1 to promote tumor proliferation and metastases is mediated via neovascularization [7,9,15]; studies suggest the angiogenic effects of IL-1 are indirect [7,23]. The indirect effects of IL-1 were illustrated in mouse Lewis lung carcinoma cells by Saijo *et al *[24]. Cells transduced with IL-1β showed no difference in proliferation rates *in vitro *but exhibited significantly increased tumor growth rates when injected into mice [24]. Additionally, there were increased microvessels and immunohistochemical staining against the endothelial cell marker, CD31, in transduced xenografts compared with the wild-type and null transfected xenografts. To further elucidate the angiogenic role of IL-1, the study measured concentrations of VEGF, macrophage-inflammatory protein-2 (MIP-2, the mouse homolog to human IL-8), and hepatocyte growth factor (HGF) proteins secreted by IL-1β-transduced Lewis lung carcinoma cells *in vitro*. *In vitro *production of VEGF and MIP-2 was significantly increased, but HGF was increased only in IL-1β-transduced xenografts. Such results demonstrate both the importance of the interaction between IL-1 and the tumor microenvironment and the indirect effects of IL-1 [24]. Previous studies in our laboratory [15] further demonstrate the indirect angiogenic properties of IL-1. The supernatant from two melanoma cell lines (PMEL and SMEL) with differential IL-1 expression (low versus high, respectively) was used in an *in vitro *endothelial cell permeability assay and results demonstrated that only IL-1 producing cell lines (SMEL and non-small-cell carcinoma, H2030) exhibited increased permeability that was inhibited by IL-1ra. Increased absorbance indicates increased endothelial cell permeability, an early step in angiogenesis (Figure 2) [15].

A study by Voronov *et al *[7] demonstrated that IL-1 is essential for angiogenesis and tumor proliferation. They found significantly increased staining for anti-Von Willebrand factor in melanoma cell-containing Matrigel plugs implanted into wild-type mice compared with IL-1β KO mice. Additionally, the angiogenic effects of IL-1 observed in the wild-type model were restored when IL-1β KO mice received plug implants containing melanoma cells with IL-1α. These studies found that IL-1 produced within the microenvironment and endogenously produced IL-1 are necessary in the neovascularization of tumors [7].

### Anti-tumor activities of IL-1ra

IL-1ra, the third member of the IL-1 family, is a naturally occurring protein that competitively blocks the IL-1RI on T lymphocytes and fibroblasts [8]. Due to its ability to block collagenase and prostaglandin synthesis within chondrocytes and synovial cells, IL-1ra (anakinra) is approved for the treatment of rheumatoid arthritis and has been showed as useful in the reversal of IL-1 mediated effects in several pathological settings [8]. IL-1ra prevented severe and lethal hypotension in rabbits administered LPS [25] and attenuated IL-1 mediated intestinal inflammation and necrosis in the immune complex-induced colitis animal model [26]. Additionally, IL-1ra blocked IL-1 induced production of colony-stimulating growth factors (CSF) by fibroblasts, lymphocytes, and monocytes in acute and chronic myelogenous leukemias [27]. Anakinra is well absorbed in humans, and its safety is well documented with few adverse reactions, making it an ideal candidate in the adjuvant therapy in cancers.

Because IL-1 promotes angiogenesis, tumor growth, and metastases, numerous studies have examined the mechanism and ability of IL-1ra to block such effects. One such study by Konishi *et al *[19] found that IL-1ra inhibited both *in vitro *and *in vivo *Vascular Endothelial Growth Factor production in colon adenocarcinoma cell lines and tumors. They noted an IL-1β dose dependent production of VEGF protein and mRNA expression in cultured colon cancer cell lines. Secretion of the protein was significantly suppressed when the cells were pretreated with IL-1ra. VEGF, IL-1β, and IL-1ra concentrations were quantified in tumor samples from patients with colon cancer, and a correlation was observed between the VEGF protein and the IL-1ra: IL-1β ratio within the tumor. Lower ratios of IL-1ra:IL-1β within the tumor correlated with increased concentrations of VEGF protein [19].

**Figure 3:**
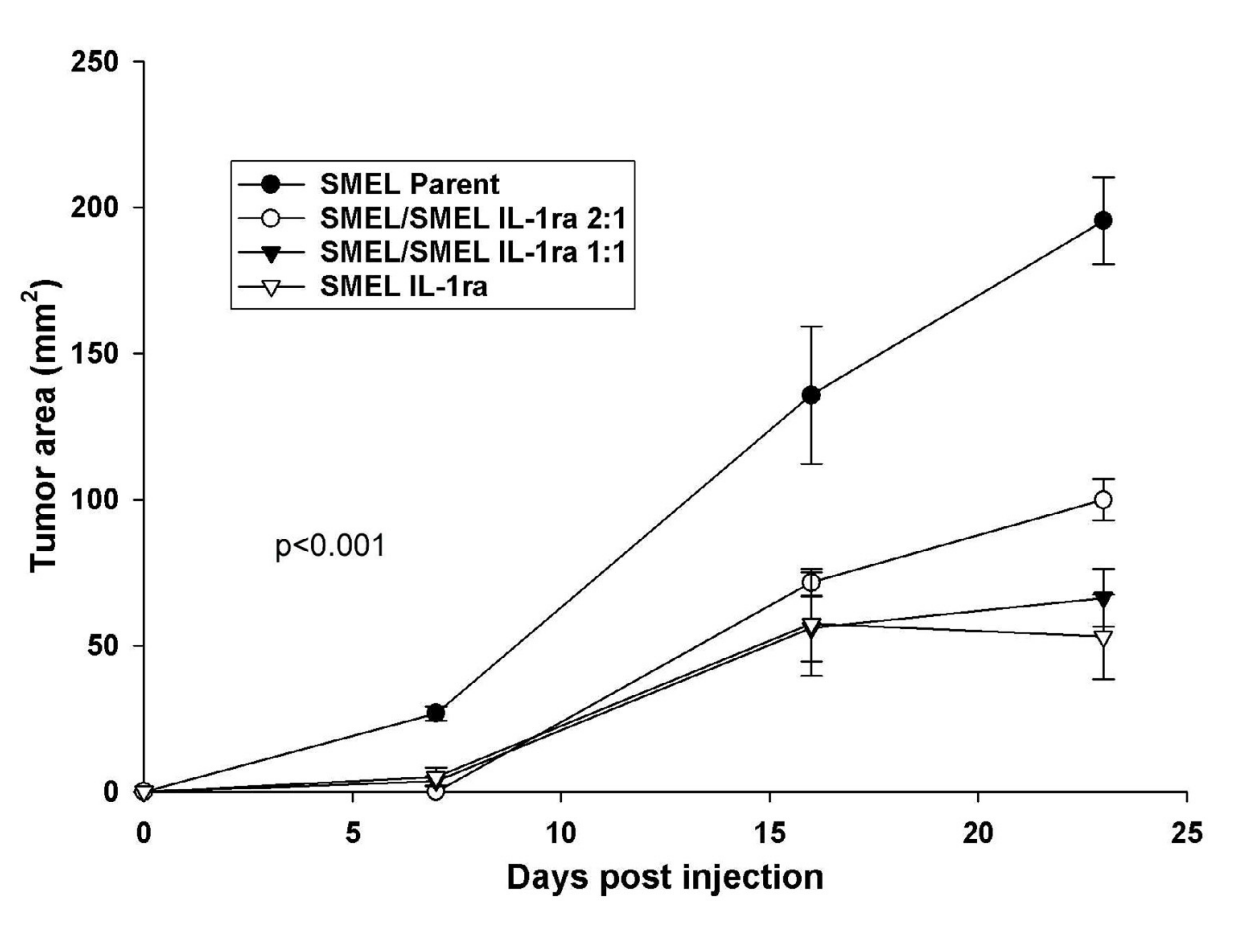
**Anti-tumor activity of IL-1ra gene transduction and over-expression in melanoma xenografts**. An IL-1 producing melanoma (SMEL) was mixed at the indicated ratios with the same cell line that was transduced to over-express IL-1ra (SMEL/IL-1ra) *ex vivo *and injected s.c. into athymic nude mice. There was significant growth inhibition at all tested ratios, indicating a marked paracrine effect of IL-1ra on tumor growth.

Our recent studies focused on the effects of constitutively produced IL-1ra on human melanoma xenografts in a murine model [28]. The specific effects of IL-1ra on tumor proliferation and metastases via the transduction of human IL-1ra into two human melanoma cell lines (PMEL and SMEL) with differential IL-1β expression (low vs. high, respectively) were illustrated. IL-1ra-transduced, null-transduced, and wild-type cells were grown *in vitro*. There were no differences in growth rates among the three groups, indicating the lack of direct autocrine effects of IL-1ra on tumor growth. Similarly, ELISA assays confirmed that IL-1ra did not alter the secretion of IL-1β in transduced cells versus the wild-type. IL-1ra behaved in a paracrine fashion, as illustrated by a mixing study in which wild-type SMEL melanoma cells and SMEL lines transduced with IL-1ra (SMEL/IL-1ra) were mixed in varying ratios *ex vivo *and then injected into athymic mice. All tested SMEL:SMEL/IL-1ra ratios resulted in significantly decreased tumor cross-sectional areas, thus demonstrating the paracrine effects of constitutively produced IL-1ra (Figure 3) [28].

**Figure 4:**
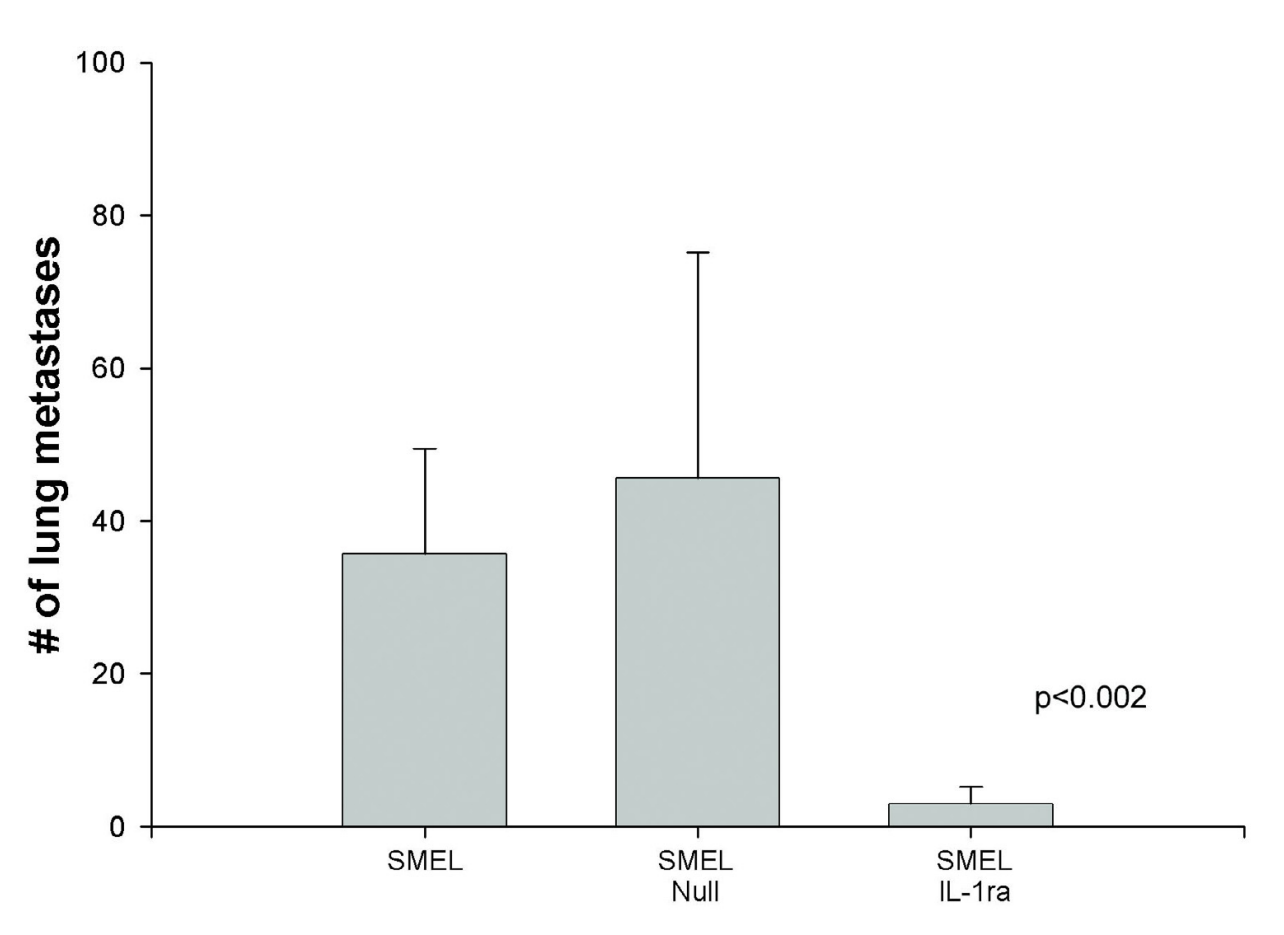
**Inhibition of lung metastases in IL-1ra over-expressing melanoma xenografts**. There was marked inhibition of lung metastases from the SMEL/IL-1ra cells compared with the null-transduced and wild-type controls. Cells were injected via tail vein, and the lungs were harvested 28 days later.

The importance of the presence of IL-1 as a tumor growth factor *in vivo *was also examined. IL-1ra-transduced and wild-type SMEL and PMEL cells were injected subcutaneously into nude mice, and only tumors which constitutively produced IL-1β (SMEL) were affected by IL-1ra transduction. There was significantly decreased tumor growth between the SMEL/IL-1ra and the null-transduced and wild-types, whereas there was no difference in the low IL-1β secreting line [28]. Additionally, SMEL/IL-1ra tumors exhibited massive necrosis within the center of the tumor which was not present in wild-type tumors, and pulmonary metastases in the SMEL/IL-1ra group were significantly fewer than in the null-transduced and wild-type groups (Figure 4) [28].

Another study evaluated the mechanism and effects of systemically administered IL-1ra on established human tumors in a murine model. Human cancer xenografts in mice receiving systemic IL-1ra administration showed decreased tumor growth and metastases only when the tumor secreted significant concentrations of IL-1β. Histologically, we failed to note any significant differences in mitotic rate between control and IL-1ra treated tumors obtained from mice. This coupled with *in vitro *experiments that also failed to demonstrate alteration in proliferation rates between IL-1ra treated and control cells, emphasizes the indirect role of IL-1 and the importance of its interaction between the tumor and microenvironment. This indirect role was further examined in experiments investigating the effect of IL-1ra on gene expression of IL-8. Iinterleukin-8, an angiogenic cytokine induced by IL-1, acts on endothelial cells to promote chemotaxis, cell retraction, and gap formation [8,29]. The results demonstrated that although IL-1ra treatment decreased IL-8  mRNA expression and protein concentration in IL-1ra sensitive xenografts, it had no effect *in vitro *[15]. Therefore, it is likely that the mechanism by which IL-1ra exerts its effects is through the tumor microenvironment rather than through direct blockade of the IL-1RI [15].

Multiple studies have investigated the necessity of IL-1 in tumor growth, metastases, and angiogenesis, which has resulted in the investigation of IL-1ra as a novel therapeutic agent in solid tumors constitutively producing IL-1. Such studies have found that IL-1ra decreases metastases and tumor proliferation *in vivo*, as well as decreasing the gene expression and production of angiogenic proteins such as VEGF and IL-8. A summary of the demonstrated anti-tumor activities of IL-1 blocking therapies is reviewed in Table 2. Based on these data, IL-1ra may prove clinically relevant in the treatment of certain cancers when used either alone or in conjunction with other chemotherapeutics.

**Table 2:** Established anti-tumor activities of IL-1 blocking methods and/or therapies based on published literature.

**Study**	**Year**	**Observation**
Voronov *et al *[7]	2003	IL-1 KO mice failed to develop solid tumors post injection of melanoma cells and exhibited significantly improved survival compared with wild type animals.
Saijo *et al *[19]	2002	Overexpression of IL-1 is associated with an aggressive/malignant phenotype.
Sawai *et al *[22]	2003	Same observation as above
Voronov *et al *[7]	2003	Same observation as above
Weinreich *et al *[28]	2003	IL-1ra-transduced xenografts exhibited decreased tumor growth and metastases in murine models.
Elaraj *et al *[15]	2006	Exogenously administered IL-1ra (anakinra) decreased tumor proliferation rate, metastases, and IL-8 and VEGF mRNA expression of xenografts in murine models.

## Novel therapeutic anti-IL-1 agents

**Figure 5:**
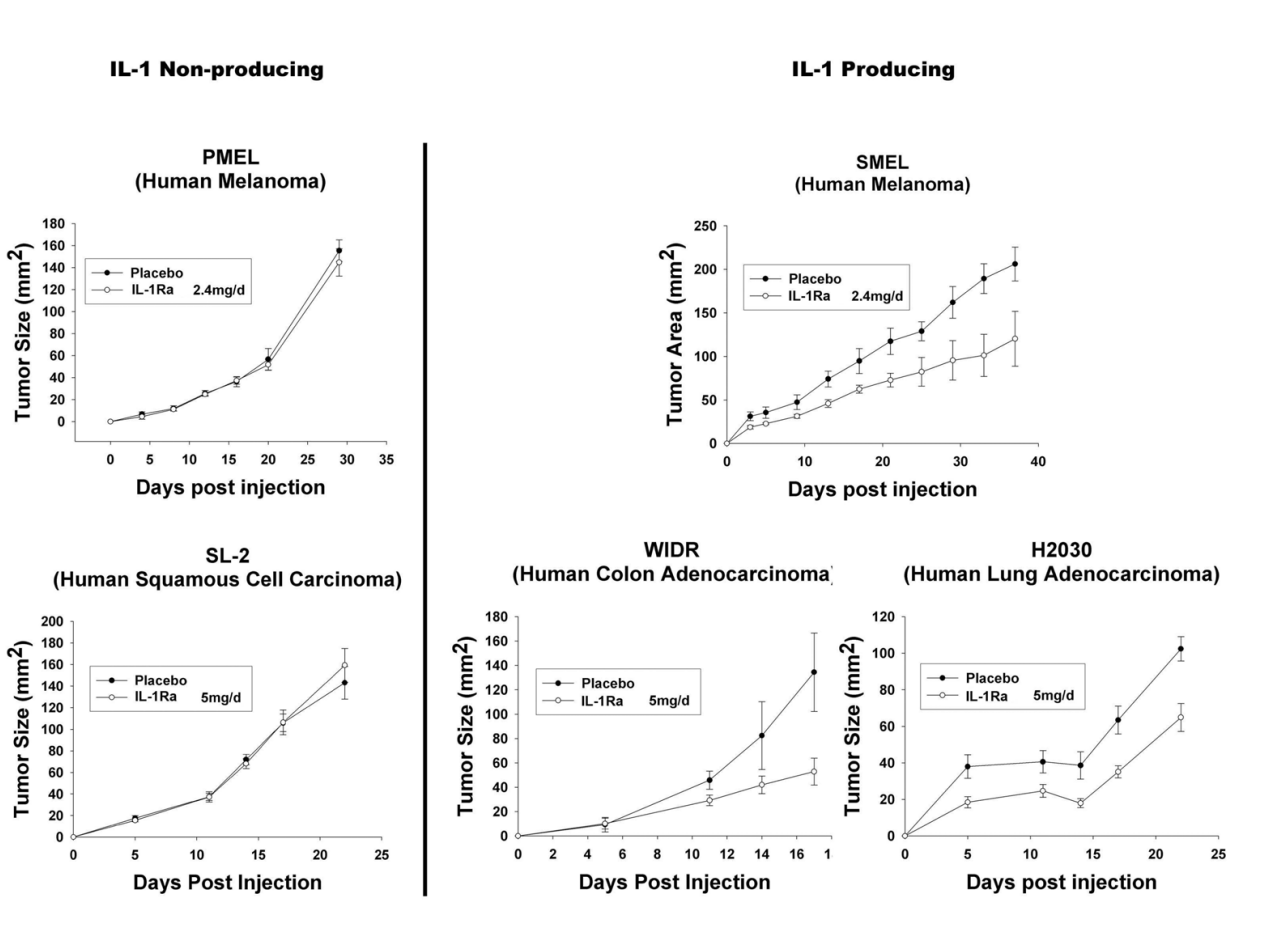
**Partial tumor growth inhibition in IL-1 but not non-IL-1 producing xenografts treated with systemic IL-1ra protein**. Two times 10^6 ^tumor cells were injected s.c. into the flanks of athymic nude mice. On the day of tumor injection, s.c. IL-1ra therapy at various doses or placebo began and was administered daily into the opposite flank from tumor injection. IL-1ra consistently resulted in a statistically significant decrease in growth rate of all three IL-1-producing tumors tested but had no effect on two non-IL-1-producing tumors.

Despite numerous studies citing the safety and potential beneficiary effects of IL-1ra, there exist questions regarding its efficiency in long term treatment of diseases such as cancer. Although anakinra has proven successful in the treatment of rheumatoid arthritis in select patients, large doses are required daily to elicit responses. IL-1 receptors are regenerated rapidly and with the exception of red blood cells, are ubiquitously expressed throughout the body. This, combined with IL-1ra's short half-life (4–6 h) presents formidable challenges to the therapeutic potential of this compound in cancer treatment. For IL-1ra to completely neutralize the tumor proliferating, angiogenic, and metastatic effects of IL-1, sustained concentrations are necessary to saturate all IL-1 receptors. Recent studies [15,28] support this hypothesis. In mice treated with IL-1ra, only partial xenograft inhibition was observed, whereas IL-1ra-transduced xenografts demonstrated near complete growth inhibition (Figures 3 &5) [15,28]. Thus, although IL-1ra has demonstrated some benefit as a cancer therapeutic, many other novel therapies remain under investigation.

### Anti-interleukin-1 monoclonal antibodies

Therapies using antibodies to IL-1β have been investigated by Neidhart *et al*. IL-1ra and anit-IL-1β monoclonal antibodies tested individually on a human rheumatoid arthritis cartilaginous matrix model decreased cartilaginous destruction by 45% and 35%, respectively [30]. Additionally, CDP-484 (Celltech), a pegylated IL-1β antibody, has demonstrated inhibitory IL-1β activities as measured by decreased neutrophil accumulation, as reviewed by Braddock *et al *[31]. In their review, Braddock *et al *report an *in vivo *study in which mice with collagen-induced arthritis were either treated with prophylactic pegylated IL-1β antibody or vehicle. Only 20% of members of the prophylactic group developed collagen-induced arthritis, whereas 90% of the control group developed the disease [31].

### Soluble IL-1RII

As mentioned previously, the IL-1 type II receptor (IL-1RII) behaves as a decoy receptor due to its lack of the cytoplasmic domain necessary for signal transduction. It therefore behaves as an inhibitor of IL-1 activity. IL-1RII occurs in membrane bound and soluble forms; the soluble IL-1RII (sIL-1RII) form naturally occurs in the synovial fluid and plasma of healthy human subjects [32,33]. A negative correlation between sIL-1RII protein concentrations and presence of chronic arthritis in patients was noted by Jouvenne *et al *in which decreased sIL-1RII protein concentrations were observed in patients with chronic arthritis [34]. These data implicate the potential anti-inflammatory effects of the soluble type II receptor. Other studies provided further evidence of the inhibitory role of sIL-1RII in the signaling and transduction of IL-1. Both *in vitro *and *in vivo *studies demonstrated that IL-1RII-transduced cells failed to respond to IL-1 [35,36]. Bessis *et al *used a collagen-induced arthritis murine model synonymous to human inflammatory arthritis to demonstrate the anti-inflammatory properties of sIL-1RtII. Human sIL-1RII-transfected keratinocytes engrated into arthritic mice decreased clinical and histological arthritis characteristics and decreased IL-6 and myeloperoixdase mRNA expression within the joints [37].

Although the IL-1RII is not responsible for IL-1 signal transduction, it does recruit the IL-1R accessory protein (IL-1RAcP) upon binding to IL-1. As previously discussed, IL-1RAcP is a necessary component for signal transduction of IL-1. The IL-1 neutralizing effects of sIL-1RII then, are twofold; it sequesters IL-1RAcP, preventing its interaction with the IL-1RI and IL-1 complex, and it acts as a decoy by binding IL-1 and preventing its binding to the signal transducing IL-1RI [12,38]. These complementary inhibitory mechanisms enhance the IL-1 blocking effects of sIL-1RII. Additionally, sIL-1RII has increased the IL-1 blocking effects of IL-1ra, and studies cite evidence that sIL-1RII and IL-1ra interact synergistically to block IL-1 activity [39,40]. Burger *et al *found that sIL-1RII and IL-1ra administered simultaneously to human keratinocytes *in vitro *resulted in decreased production of prostaglandin E_2 _and metalloproteinases compared with either one treatment alone [40]. Based on such studies, combined novel therapeutics may be more effective in blocking the deleterious effects of IL-1 than individual treatments.

### Interleukin-1β-converting enzyme (ICE) inhibitors

IL-1β-converting enzyme (ICE), also known as caspase-1, is a protease necessary in the conversion of inactive IL-1β to the mature pro-inflammatory form and recently has been investigated as a novel therapeutic agent with IL-1β blocking potential. Additionally, ICE is responsible for the conversion of the pro-IL-18 form to its mature active form. Thus the advantages of employing ICE inhibitors are twofold: they diminish the actions of inflammatory cytokines IL-1 and IL-18 [41]. Due to the oral availability of ICE inhibitors, they are advantageous compared with injectable protein products such as IL-1ra. Patients prefer oral medications over injected ones, and orally administered drugs may result in better compliance and reduced cost [42]. The safety of ICE inhibitors remains under surveillance, and animal studies suggest this therapeutic is well-tolerated. Such studies found that genetically ICE-deficient mice did not appear to be at increased risk of developing infection or malignancies [43,44]. Furthermore, Rudolphi *et al *report that pralnacasan, the first orally bioavailable ICE inhibitor tested in humans, was well-tolerated in healthy subjects in a phase I/IIa clinical trial [45]. The effectiveness of pralnacasan was also investigated by Rudolphi *et al *in two osteoarthritis murine models where pralnacasan significantly decreased histopathological joint cartilage destruction. A phase II clinical trial involving pralnacasan for the management of osteoarthritis is underway [45]. ICE inhibiting agents such as pralnacasan and the more recent VX-765 demonstrate promise as anti-inflammatory therapeutics in that they are orally available and they elicit the anti-inflammatory response via the inhibition of the activities of both IL-1 and IL-18.

### Cytokine traps

As discussed previously, IL-1 first binds to the IL-1RI, then recruits a second binding protein, the IL-1RAcP. IL-1 binds more avidly to this complex than the individual receptor, which is the basis upon which cytokine traps were founded [46]. Cytokine traps are produced by eukaryotic cells in culture and are soluble recombinant proteins consisting of cytokine cell-surface receptor domains bound inline to the Fc portion of human IgG1. These dimeric molecules avidly bind the cytokine of interest and have been developed for TNFα, IL-4, IL-6, and IL-1 [46]. Both *in vitro *and *in vivo *studies by Economides *et al *determined that the IL-1 trap more effectively blocked IL-1 and its effects than did IL-1ra. *In vitro *experiments measured the relative efficacies (IC_50_) of the IL-trap and IL-1ra; in the presence of IL-1β (4 pM), the IC_50 _of IL-1 trap versus IL-1ra were 2 pM and 70 pM, respectively [46]. Additionally, the IL-1 trap avidly bound to IL-1α as well as IL-1β, an advantage over monoclonal antibodies against a single form of IL-1. *In vivo *studies found that IL-1 traps injected subcutaneously into mice 24 h prior to injection with IL-1β resulted in complete inhibition of IL-6 production, a cytokine induced by IL-1. This single dose also blocked the effects of a subsequent IL-1β injection 24 hours later, thus highlighting the ability of the IL-1 trap to exert its blocking activities over a prolonged amount of time. In contrast, IL-1ra did not inhibit the activities of IL-1 when administered at a dose 15-fold greater than the IL-1 trap dose [46].

To date, this study has demonstrated that the IL-1 trap has more successfully inhibited IL-1 activity than any other published study involving IL-1 blocking therapies, and based on entropy principles, it has been suggested that the IL-1 trap may bind IL-1 more avidly than even the IL-1RI itself [46]. Additionally, the ability of IL-1 traps to bind multiple cytokines such as IL-1α and IL-1β, coupled with its prolonged half life *in vivo *render it a rapidly emerging novel therapeutic agent in the inhibition of IL-1 activity. Results of a completed phase II study involving the treatment of 200 patients with weekly subcutaneous doses of IL-1 trap have been reported [31]. In this clinical trial, IL-1 traps appeared to be safe, well-tolerated, and elicited no abnormal clinical laboratory values or evidence of increased number of infections, thus supporting further investigations of this protein.

## Conclusion

Elevated IL-1 concentrations have been identified in numerous types of solid tumors in which the prognoses is markedly worse. This pleiotropic cytokine promotes tumor proliferation, angiogenesis, and metastases via its autocrine and paracrine effects within the tumor itself as well as the microenvironment. Our recent studies highlight the potential beneficial effects of both constitutively produced and exogenously administered IL-1ra, while numerous other IL-1 blocking therapeutics including anti-IL-1 monoclonal antibodies, soluble IL-1RII, ICE inhibitors, and IL-1 cytokine traps have shown promise in the treatment of rheumatoid arthritis. The goal is to apply such novel treatments either alone or in conjunction with more traditional approaches towards the inhibition of IL-1 in the treatment of cancer.

## Competing interests

The author(s) declare that they have no competing interests.

## Authors' contributions

AML drafted and edited the manuscript. SV participated in data collection and analysis and edited the manuscript. HX participated in data collection and analysis. HRA conceived the study design and helped draft and edit the manuscript. All authors read and approved the manuscript.
